# Candidate genes associated with low temperature tolerance in cucumber adult plants identified by combining GWAS & QTL mapping

**DOI:** 10.1007/s44154-024-00191-9

**Published:** 2024-12-11

**Authors:** Caixia Li, Shaoyun Dong, Diane M. Beckles, Xiaoping Liu, Jiantao Guan, Zaizhan Wang, Xingfang Gu, Han Miao, Shengping Zhang

**Affiliations:** 1 State Key Laboratory of Vegetable Biobreeding, Institute of Vegetables and Flowers, Chinese Academy of Agricultural Sciences, Beijing, 100081 China; 2 Department of Plant Sciences, University of California, One Shield Avenue, Davis, CA 95616 USA

**Keywords:** Cucumber, Low temperature tolerance, GWAS, QTL, Candidate gene analysis

## Abstract

Fruit quality and yield are reduced when cucumber (*Cucumis sativus* L.) plants are exposed to low temperature (LT) stress, yet, the inheritance and genes linked to cold tolerance in adult plants have not been reported yet. Here, the LT-tolerance of 120 cucumber accessions representing four ecotypes were evaluated by GWAS, and also, in 140 recombinant inbred lines (RILs) derived from a biparental cross. Plants were exposed to naturally occurring LT environments in a plastic greenhouse, in winter 2022, and 2023, and a low temperature injury index (LTII) was employed to evaluate plant performance. Genetic analysis revealed that the LT-tolerance evaluated in the adult cucumber plants was a multigenic quantitative trait, and that 18 of the 120 accessions were highly LT tolerant by our LTII assessment. Two loci (*gLTT1.1* and *gLTT3.1*) exhibited strong signals that were consistent and stable in two environments. In addition, two QTLs—*qLTT1.2* on chromosome (Chr.) 1, and *qLTT3.1* on Chr. 3, were discovered in all tests using RIL population derived from a cross between LT-sensitive ‘CsIVF0106’, and LT-tolerant ‘CsIVF0168’. *qLTT1.2* was delimited to a 1.24-Mb region and *qLTT3.1* was narrowed to a 1.43-Mb region. Interestingly, a peak single nucleotide polymorphism (SNP) at *gLTT1.1* and *gLTT3.1* was also found in *qLTT1.2* and *qLTT3.1*, respectively. These loci were thus renamed as *gLTT1.1* and *gLTT3.1*. In these regions, 25 genes were associated with the LT response. By identifying differences in haplotypes and transcript profiles among these genes, we identified four candidates: *CsaV3_1G012520* (an ethylene-responsive transcription factor) and *CsaV3_1G013060* (a RING/U-box superfamily protein) in *gLTT1.1*, and two RING-type E3 ubiquitin transferases at *CsaV3_3G018440* and *CsaV3_3G017700* in *gLTT3.1* that may regulate LT-tolerance in adult cucumber. Interestingly, the accessions in which the LT-tolerant haplotypes for two loci were pyramided, displayed maximally high tolerance for LT. These findings therefore provide a solid foundation for the identification of LT-tolerant genes and the molecular breeding of cucumber with LT-tolerance.

**Supplementary Information:**

The online version contains supplementary material available at 10.1007/s44154-024-00191-9.

## Introduction

Cucumber (*Cucumis sativus* L.) is considered one of the most critical vegetables and is extensively cultivated worldwide. The global production of cucumber has reached 77.3 million tons, with 81.6% of this production originating from China (FAOSTAT, 2022). Due to the tropical origins, cucumber exhibit sensitivity to low temperatures (LT) throughout all stages of their life cycle (Cabrera et al. 1992). The optimal growing temperature for cucumber is 18℃ ~ 30℃ (Smeets and Wehner 1997), therefore when temperatures fall below 10℃ in early spring, late autumn, or early winter, which occurs with increasing frequency in recent years, production decreases (Smeets and Wehner 1997). In winter, abrupt drops in temperature during cultivation leads to visible defects, which collectively results in a loss of quality, and more catastrophically, yield (Smeets and Wehner 1997).

Most studies on cucumber response to LT stress focus on germinating seeds or seedlings. Therefore, the methods used for evaluating stress response are related to the physiology of these developmental stages. For seed germinability, relative germination-percentage, -energy, -indices, relative radicle length, and days to 50% seed germination are assessed. Of these parameters, relative germination percentage is often used (Kłosińska et al. 2013; Song et al. 2018; Yagcioglu et al. 2019). At the seedling stage, the rate of electrolyte leakage is most widely used LT-tolerance indicator (Miao et al. 2013; Qi et al. 2011), but chlorophyll content and photosynthetic intensity have been studied (Yu et al. 2002; Kozik and Wehner 2008; Dong et al. 2019). However, no reports on QTL mapping for LT-tolerance during adult cucumber plants was reported, therefore a uniform standard for assessing this trait has not yet been identified.

Few studies detailing the genetic inheritance of LT-tolerance in cucumber and all data come from germinating seeds and seedling-staged plants. Tolerance to LTs in germinating seedlings have been reported to be polygenic (Kłosińska et al. 2013; Song et al. 2018; Dong et al. 2019). There are also conflicting reports as maternal inheritance and control by a single gene have been suggested (Chung et al. 2003; Kozik and Wehner 2008), but recent studies point to control by multiple genes (Dong et al. 2019; Kozik et al. 2012). However, the genetic basis for LT response in adult cucumber plants have not been reported.

Due to advances in genomic tools, it is feasible to genetically dissect significant agronomic traits in cucumber, and QTLs underlying LT-tolerance have been successfully identified. Eight loci related to tolerance at germination were found on Chr.4, 6, and 7 (Dong 2017). Major effect QTLs, i.e., *qLTG1.1* or *qLTG1.2*, as well as minor effect QTLs, like *qLTG2.1* and *qLTG4.1*, were also discovered (Song et al. 2018; Yagcioglu et al. 2019; Li et al. 2022b). One marker, SSR07248, was associated with a major LT-tolerance locus on Chr.6 in seedlings, by scoring the population with a cold injury index (CII) (Li et al. 2015). Meanwhile, three loci—*qCT-3–1*/*3–*2/*3–3* on Chr.3, *qCT6.1* on Chr.6, and *qCT-7–1* on Chr.7, were also detected using a CII (Li et al. 2022a; Wang 2014).

Further dissection of QTL regions has identified genes that regulate plant response to LT. Two candidate genes: *Csa6G445210*, encoding an auxin response factor, and, *Csa6G445230*, encoding an ethylene-responsive transmembrane protein (*Csa6G445230*) were associated with LT-tolerance in cucumber seedlings (Dong et al. 2019). Functional validation of the cucumber *CsWRKY46* gene in Arabidopsis provided evidence that it enhances cold tolerance (Zhang et al. 2016). Three genes were reported associated with LT-tolerance in cucumber (Yan et al. 2022; Qi et al. 2022; Dong et al. 2023). The suppression of *CsGPA1* decreases LT-tolerance in cucumber, and that the *CsGPA1–*cold regulated *CsCOR413PM2–Ca*^*2*+^ axis, in turn, regulates the expression of *CsCBF* during cold stress (Yan et al. 2022). Qi et al. (2022) demonstrated that overexpressing heat-shock transcription factor *CsHSFA1d,* activated the *ICE-CBF-COR* signaling cascade via jasmonic acid (*JAZ5*), and enhanced cucumber cold tolerance. Dong et al. (2023) suggested that allelic variants of *CsSGR* enhances LT-tolerance in cucumber by a) interacting with, and inhibiting the action of chlorophyll degradation enzymes and b) by reducing harmful ROS production. However, genes related to LT-tolerance in adult-staged cucumber plants have yet to be discovered.

Genome-wide association studies (GWAS) have been extensively utilized in cucumber to pinpoint numerous intricate quantitative traits, including responses to abiotic stressors. Seven loci for salt tolerance were identified in 220 cucumber accessions (Liu et al. 2022a). Seven loci significantly associated with heat stress response in the seedlings of 96 accessions were identified by GWAS (Wei et al. 2019). Heat tolerance in adult plants were evaluated using 88 accessions and five loci for heat stress response were identified (Wang et al. 2023b). LT-tolerance at the germination stage was evaluated in 151 cucumber accessions and seven loci associated with LT-tolerance were examined. Dissection of one of these loci showed that *pentatricopeptide repeat-containing protein* (*CsPPR*) was a negatively regulator of cucumber LT-tolerance (Li et al. 2023b). Five LT-tolerance loci were identified for cold tolerance in 87 cucumber accessions (Wang et al. 2019b). This study was subsequently expanded to include 173 cucumber accessions which led to an additional three identified loci, that were repeatedly detected across two years and two independent experiments (Dong et al. 2023). However, no studies were reported of LT-tolerance at cucumber adult stage using GWAS.

Our aim in this work is to identify genes that could provide tolerance to LT in adult cucumber plants to bridge the current gap in this knowledge. Much progress has been made in identifying LTT genes at juvenile stages, but cucumber, like all tropical species, is sensitive to cold stress at each stage of development. LT injury at the adult stage has a direct effect on reproductive processes and directly influences yield. Identifying and introgressing multiple genes could provide robust LT-tolerance throughout the lifecycle is thus an important goal. To address this, we conducted a GWAS involving 120 accessions. Additionally, we analyzed a recombinant inbred line (RIL) population derived from a cross between the LT-sensitive line 'CsIVF0106' and the LT-tolerant line 'CsIVF0168'. These approaches were employed to identify loci related to LT tolerance and to investigate potential candidate genes by functional annotation, haplotypes and transcript profile analysis. Our long-term objectives of this work are to (i) identify multiple sources of genetic variation for LTT in cucumber adult plants; (ii) screen LT-resistant accessions for genetic improvement; and (iii) dissect the molecular mechanisms underlying LT response. Collectively, this knowledge will facilitate the development of LTT cucumber varieties through marker-assisted selection in breeding programs.

## Results

### Genetic diversity of LT-tolerance among the cucumber accessions

**Fig. 1:**
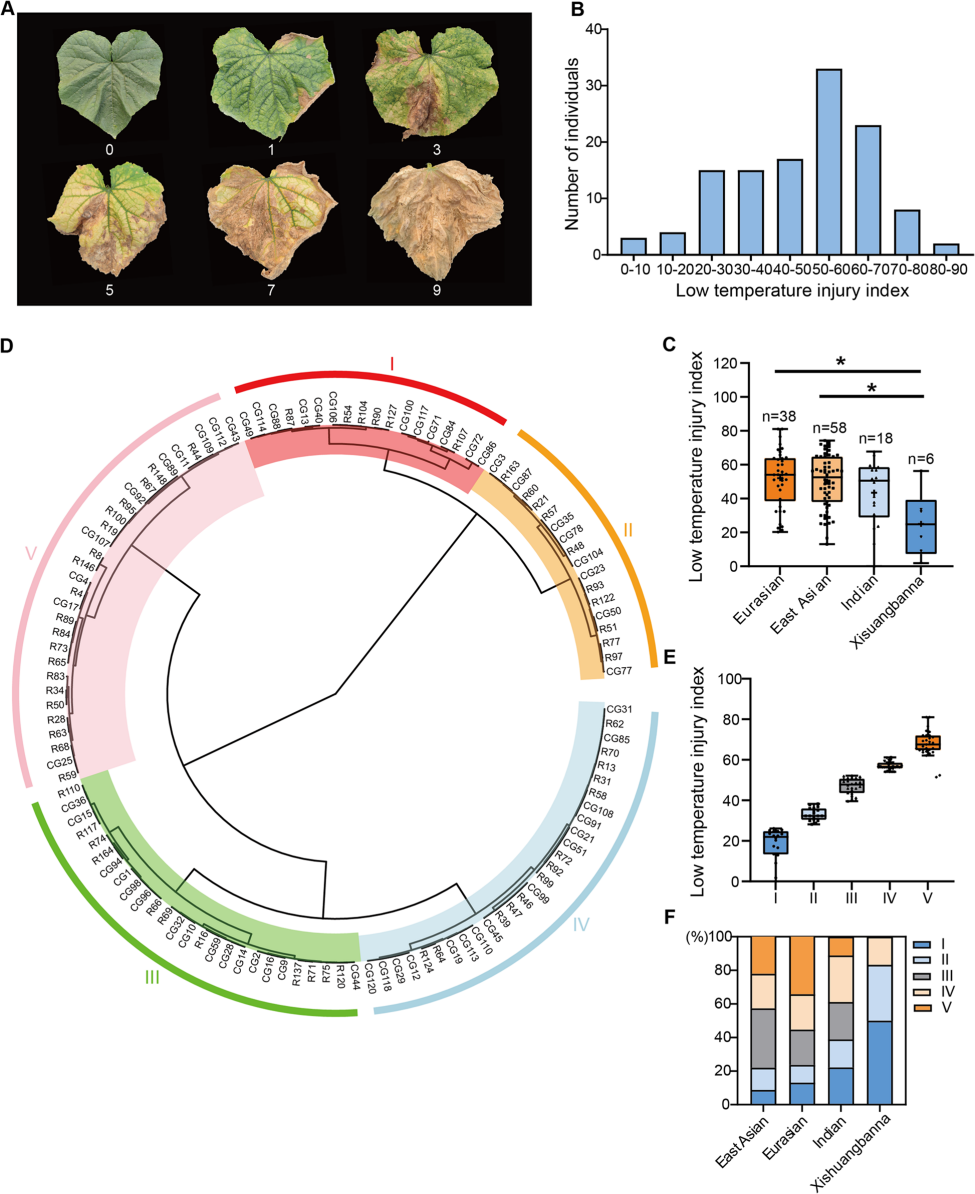
Phenotypic characterization of LT-tolerance and cluster analysis of 120 cucumber accessions at the adult stage. **A** The grades utilized in determining the Low Temperature Injury Index (LTII) are established through evaluating the extent of yellowing and dryness in the fourth to sixth functional leaves of each plant from top to bottom. Grade 0: No symptoms visible, leaves were dark-green; Grade 1: leaves were green; slight withering at the leaf’s edge; Grade 3: leaves were light-green; withered area was < 50%; Grade 5: leaves were yellow; withered area was 50–75%. Grade 7: leaves were dark-yellow; withered area was > 75%; Grade 9: All leaves were withered, and plants died. **B** A bar chart indicating the frequency distribution of LTII. **C** The box plot indicates the distribution of LTT across the four ecotypes (Eurasian, East Asian, Indian and Xishuangbanna). **D** The 120 accessions grouped based on LTII. I, II, III, IV and V indicates that clusters of accessions ranging from high to low LTT, respectively. **E** A box plot depicting the LTII of cucumber accessions, organized into Clusters based on their tolerance. **F** The relative proportion of the clustered accession (I -V), within the Eurasian, East Asian, Indian and Xishuangbanna

The LT-tolerance of 120 cucumber accessions was assessed using LTII in 2022_W and 2023_W (Fig. 1A). The plants were subjected to naturally occurring LT stress at approximately 40 days after sowing in 2022 (average 12.9 ℃) and in 2023 (average 14.4 ℃) (Table S1; Fig. S1). The accessions showed obvious yellowing and dryness of leaves at 96 days after sowing in 2022_W and at 102 days after sowing in 2023_W, respectively. The LTII of the accessions ranged from 0 to 81, and from 22.5 to 81, with average LTII values of 22.98 and 15.56 in 2022_W and 2023_W, respectively (Table S2). The coefficient of variation for each year was 59.45% and 27.53%, respectively, indicating significant variation in the LTIIs among the cucumber accessions (Table S2). The LTII showed a continuous variation from tolerant to sensitive phenotypes among the 120 accessions (Fig. 1B), suggesting that LT-tolerance is quantitative trait, influenced by the action of multiple genes.

### Cluster analysis of LT-tolerance in cucumber adult plants

To further analyze the LTT across the population, the 120 cucumber accessions were categorized into four ecotypes, i.e., Eurasian (consisting of 38 lines), East Asian (comprising 58 lines), Indian (including 18 lines) and Xishuangbanna (comprising 6 lines) (Table S3). The Xishuangbanna ecotypes showed a higher LT resistance compared to the accessions from Eurasian and East Asian (Figs. 1C). The LTII analysis resulted in the classification of the 120 cucumber accessions into five distinct clusters (I, II, III, IV, V) using the Ward method, with a Euclidean distance threshold of 700. Cluster I comprised 18 accessions characterized by high tolerance to LT; Cluster II consisted of 18 LT-tolerant accessions; Cluster III included 26 accessions showing mid-range LT-tolerance; while the 28 accessions in Cluster IV and the 30 accessions in Cluster V showed LT-sensitivity and extreme highly LT-sensitivity, respectively (Fig. 1D, Table S4). The difference in LTII among the five clusters were significant (*P* < 0.05) (Fig. 1E). Three ecotypes, i.e. those from East Asia, Eurasia and India, included multiple clusters from I to V but differed in the relative proportions of each (Fig. 1F). The Xishuangbanna types were mainly grouped into the LT tolerant Clusters, i.e., I, and II, with a relatively smaller proportion in Cluster IV (Fig. 1C,1F), which was consistent with the result in Fig. 1C.

### GWAS analysis of LT-tolerance at adult stage

**Fig. 2:**
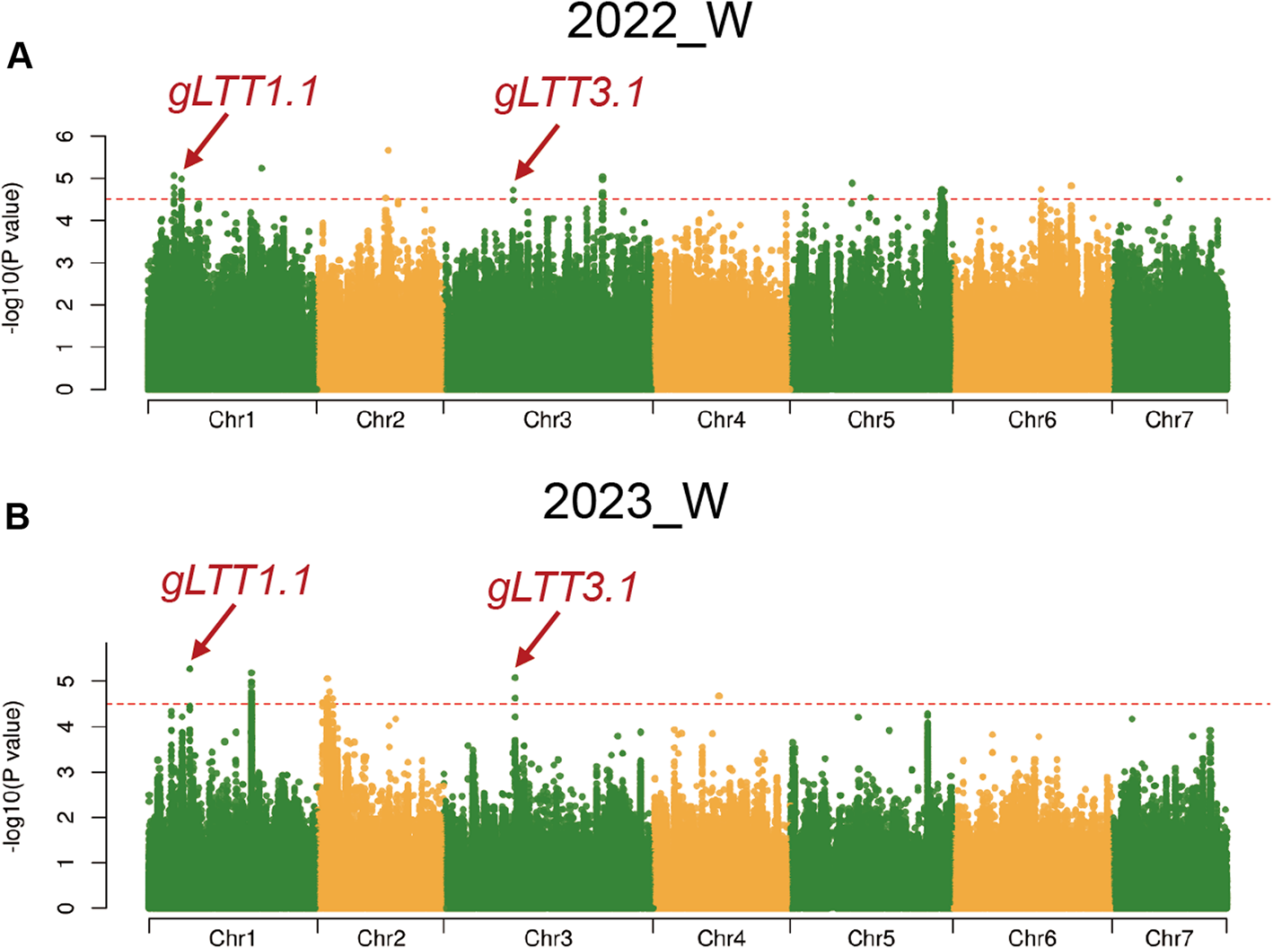
Genome‐wide association studies (GWAS) identify *gLTT1.1* and *gLTT3.1* loci. Manhattan plots of GWAS for LTII measured in (**A**) 2022_W and (**B**) 2023_W. The red horizontal dashed lines represent the GWAS significance threshold of (-log10(*P*) = 4.5). Arrows indicate the position of peak SNPs

GWAS analysis utilized LTII values alongside re-sequencing data and FaST-LMM. When the threshold was set as 4.5, SNP loci distributed on all seven chromosomes were detected in the two environments tested. Of these loci, two distributed on Chr.1 and Chr.3 were repeatedly detected in 2022_W and 2023_W, and were subsequently named *gLTT1.1* and *gLTT3.1*, respectively (Fig. 2, Table S5). These two loci were identified as novel and consistently associated with LT-tolerance at cucumber adult stage.

### QTL mapping of LT-tolerance

In our lab, a RIL population was constructed using ‘CsIVF0106,’ (derived from the LT-sensitive Cluster IV accession), and ‘CsIVF0168’ (from the LT-tolerant Cluster II accession), as parentals. The RIL population, their parents, and the F_1_ were planted in a plastic greenhouse in 2021 and 2022, then exposed to a naturally-occurring LT stress in 2021 (average 13.5 ℃) and 2022 (average 12.9 ℃) (Table S1; Fig. S1). The fourth, fifth and sixth leaf from the top of the ‘CsIVF0106’ plant were completely dried with LTIIs of 50.85 and 79.8 in 2021_W and 2022_W, respectively. However, ‘CsIVF0168’ grew normally; the leaves were green and showed no signs of withering, and the LTIIs were 22.05 and 19.8 respectively—less than half those of ‘CsIVF0106’. The LTII of the F_1_ population was 33.3 and 27.0, and the symptoms were similar to the resistant parent ‘CsIVF0168’ (Table S6, Fig. S1A). The frequency distributions of the RILs showed a clear bimodal distribution (Fig. S1B and 1C), suggesting that LT-tolerance is a quantitative trait in adult cucumber plants.

**Fig. 3:**
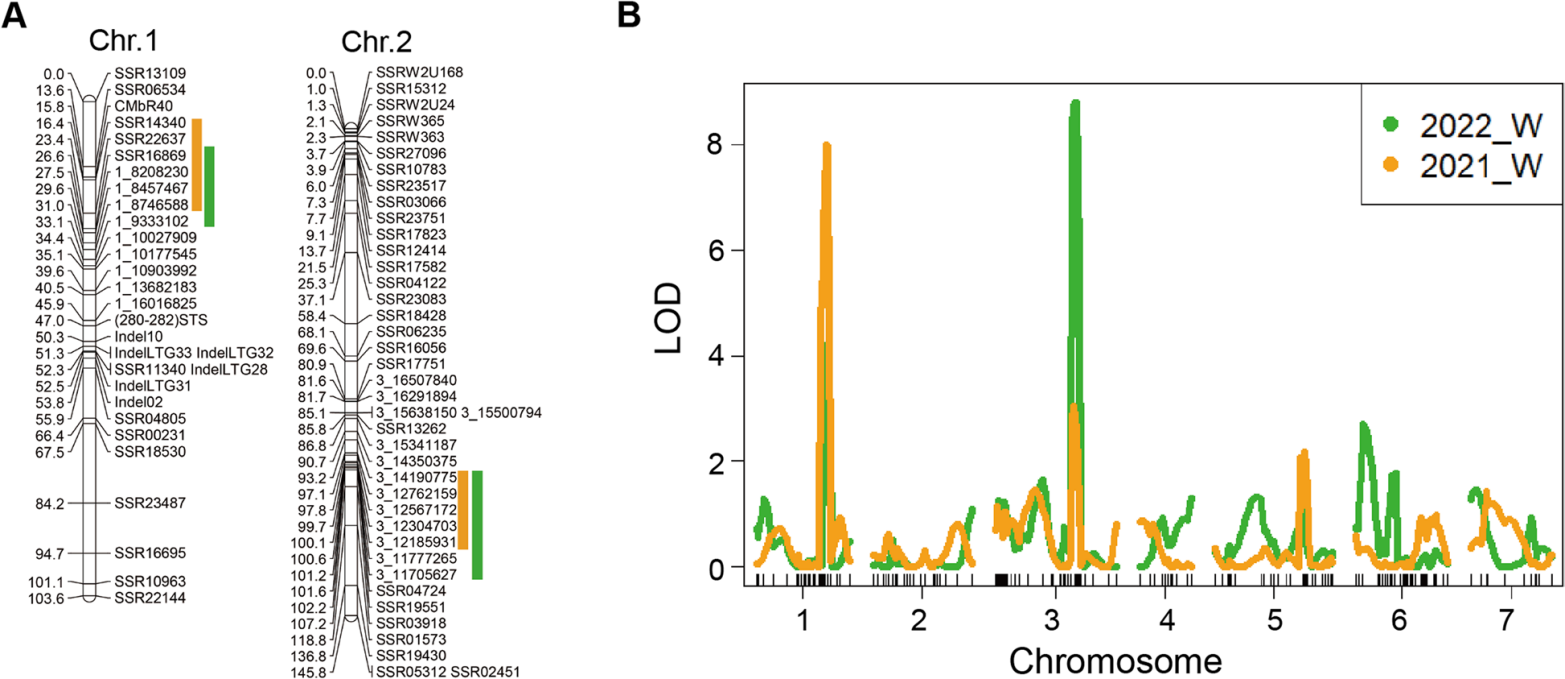
QTL mapping of LT-tolerance at cucumber adult stage.** A** The genetic maps of Chr.1 and Chr.3 respectively. **B** The X-axis represents the genetic position of each chromosome; the Y-axis represents the LOD scores. The green and orange lines represent experiments done in 2021_W and 2022_W, respectively

**Fig. 4:**
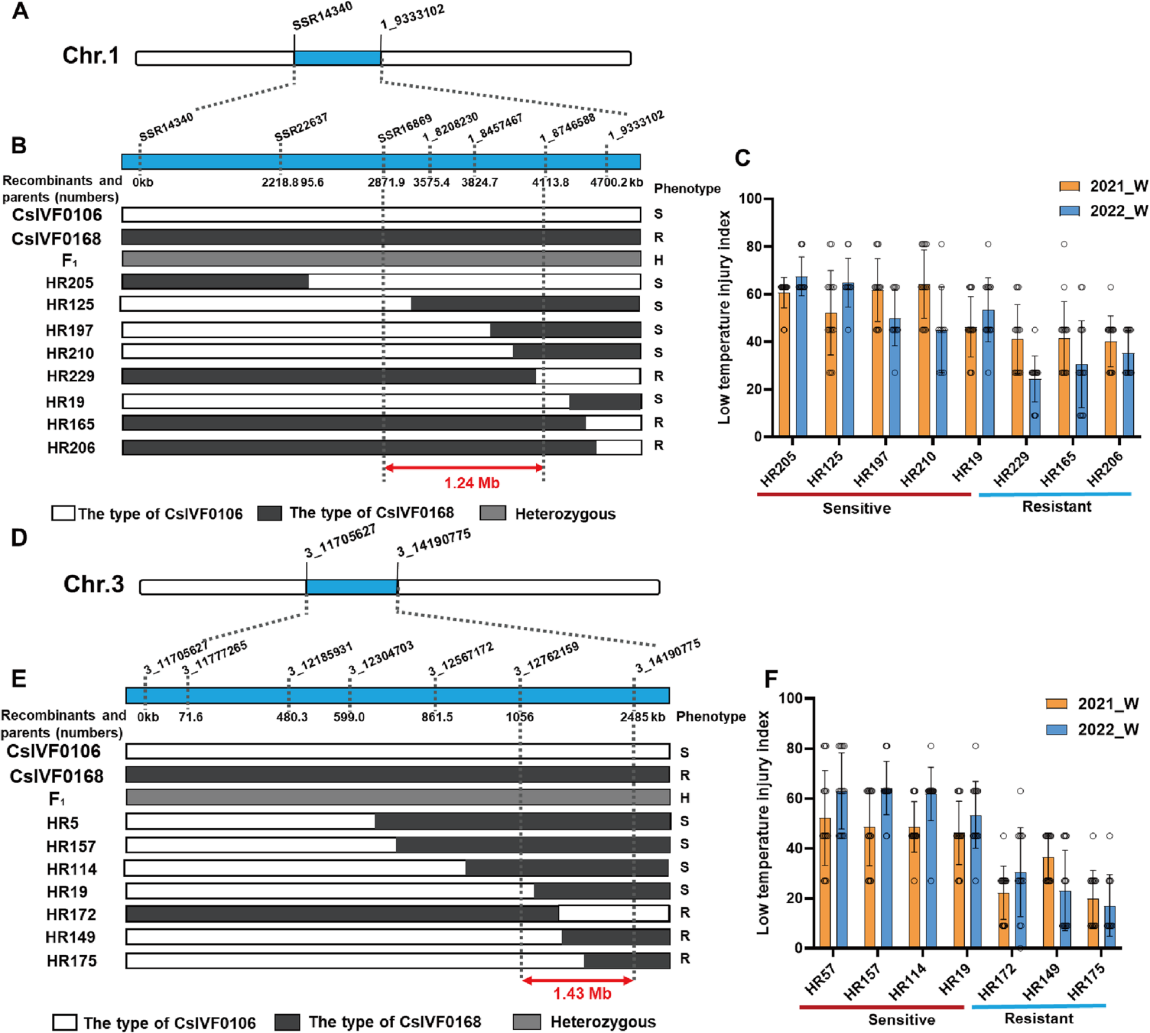
Fine mapping of *qLTT1.2* and *qLTT3.1* using phenotypic data collected in 2021_W and 2022_W. **A** The QTL map of *qLTT1.2* delimited by the two key flanking markers (**B**) Fine mapping of *qLTT1.2* with seven InDel or SSR markers to a 1.23 Mb region on Chr.1. On the map, the physical distances between the InDel markers are depicted relative to their positions in the '9930' cucumber genome. Homozygous fragments from 'CsIVF0106' and 'CsIVF0168' are denoted by white and black bars, respectively, while gray bars indicate heterozygous regions. **C** The phenotypic data (LTII) of sensitive and resistant individuals from the RIL population. **D** The QTL map of *qLTT3.1* showing the two flanking markers*.* **E** Seven InDel markers were employed to fine map the *qLTT3.1* locus to a region spanning 1.43 Mb on Chr.1. The physical distance between the InDel markers are indicated based on their position on ‘9930’; white, black and gray bars are as described in (**B)**. **F** The phenotypic data of RIL population, i.e., recombinants ‘HR57’, ‘HR157’, ‘HR114’, ‘HR19’, ‘HR172’, ‘HR149’ and ‘HR175’

A linkage map with 156 markers was previously developed using a RIL population (Shi et al. 2018). Further, based on the biparental sequence information, 18 InDels markers were developed, and a new linkage map of Chr.1 and Chr.3 was established (Fig. 3A). The phenotypic data (Table S7) were integrated with the genetic map from the RILs population to identify QTLs linked to LT- tolerance. Table S9 displays the chromosome location, QTL peak position, peak logarithm of odds (LOD) scores, and the proportion of total phenotypic variance explained (R^2^). The results indicated that three QTLs i.e., *qLTT1.2*, *qLTT3.1*, and *qLTT5.1* were detected in both years, with a LOD score of 2.5 (Table S8; Fig. 3B). Of these, *qLTT1.2* and *qLTT3.1* were also detected in both locations. For each year, the LOD scores of *qLTT1.2* were 2.85 and 6.34, and the phenotypic variation was 7.83% and 20.65% of, respectively (Table S8). A 4.7 Mb region was identified using markers SSR14340 and 1_9333102 (Fig. 4A). To further reduce the preliminary mapping region of *qLTT1.2*, four InDel markers were developed using the genomic sequence of ‘CsIVF0106’ and ‘CsIVF0168’ (Fig. 4B). Using a set of sensitive recombinants, i.e., ‘HR205’, ‘HR125’, ‘HR197’, ‘HR210’, and ‘HR19’ and, those that are resistant, i.e., ‘HR229’, ‘HR169’, and ‘HR206,’ individuals (Fig. 4C), *qLTT1.2* was further delimited to a region with a physical distance of 1.24 Mb, between markers SSR16869 and 1_8746588. For *qLTT3.1*, the LOD scores were 6.34 and 3.22, and they explained 16.54% and 8.88% of the phenotypic variation respectively, in a 2.49 Mb region delimited by markers 3_ 11,705,627 and 3_14190775 (Fig. 4D). To further reduce the *qLTT3.1* interval, seven InDel markers were generated using the genomic sequence of ‘CsIVF0106’ and ‘CsIVF0168’ (Fig. 4E). Integrating the region defined by markers with the phenotypic of sensitive recombinants (Fig. 4F), i.e., ‘HR57’, ‘HR157’, ‘HR114’, ‘HR19’, and resistant recombinants, i.e., ‘HR172’, ‘HR149’, ‘HR175’, *qLTT3.1* was mapped to a 1.43 Mb region flanked by markers 3_12762159 and 3_14190775.

### Candidate gene analysis for the identified loci

To pinpoint dependable candidate genes, our attention was directed towards the *gLTT1.1* and *gLTT3.1* loci. We conducted our analysis using a 50 kb region upstream and downstream of each SNP (Purcell et al. 2007)**.** By comparing the SNP position with the QTLs, we found that the GWAS-identified *gLTT1.1* locus colocalized with *qLTT1.2* identified by QTL mapping; this locus we will refer to as *gLTT1.1*. Similarly, *gLTT3.1* identified by GWAS colocalized with the QTL-mapped *qLTT3.1* and which will be referred to as *gLTT3.1*. These data indicate that the identified loci are reliable. Loci *gLTT1.1* spanned 6,460,155 bp to 8,746,588 bp on Chr.1 and *gLTT3.1* from 12,762,159 bp to 14,190,775 bp on Chr.3, and these were the region analyzed.

Candidate genes associated with abiotic stress within *gLTT1.1* and *gLTT3.1* were further compared for sequence and expression difference. These comparisons were done on two groups of accessions varying in cold response. The cold tolerant group consisted of accessions, ‘CG84’ (from Group I), and, ‘R51’ and ‘R77’ from Group II. These were compared to cold sensitive accessions: ‘CG110’, ‘CG31’, and ‘CG19’ from Group IV, and ‘R59’ from Group V.

**Fig. 5:**
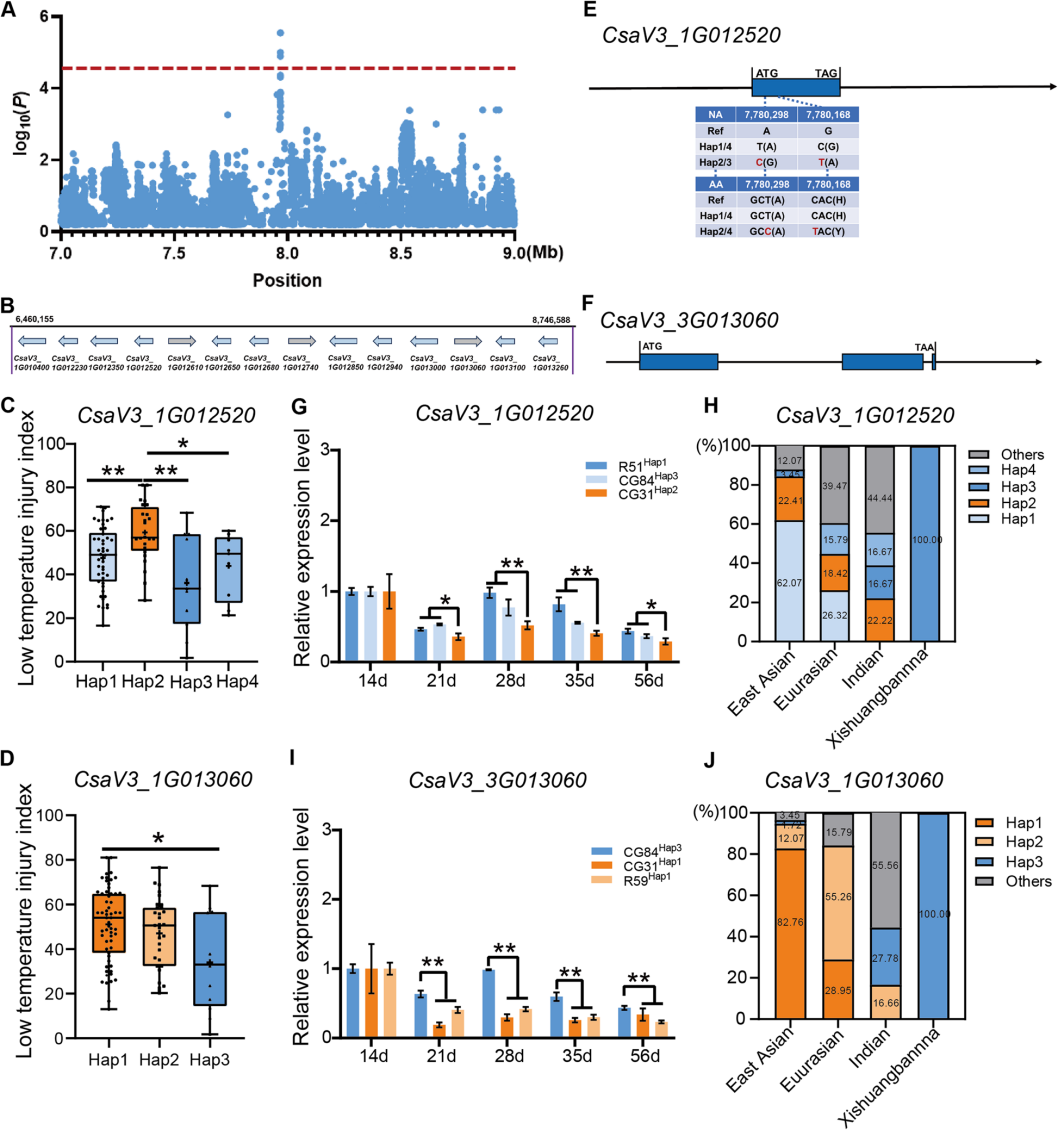
Identification of low-temperature associated gene *CsaV3_1G012520* and *CsaV3_1G013060* in *gLTT1.2* locus at cucumber adult stage. **A** Local Manhattan plot of the *gLTT1.1* locus (top), the red horizontal dashed line indicates the GWAS significance threshold of (-log10(*P*) = 4.5). **B** Fourteen genes associated with abiotic stress were identified in the *gLTT1.2* candidate region. Each gene is represented by an arrow, indicating its directionality. The box plots show the LTII distributions of accessions carrying distinct haplotypes of the *CsaV3_1G012520* (**C**) and *CsaV3_1G013060* (**D**) genes. **E** Sequence analysis of *CsaV3_3G012520*. The single base substitution (C/T) would cause a (His → Tyr) mutation, and another base substitution (T/C) caused a nonsense mutation. **F** Sequence analysis of *CsaV3_1G012520* showed that there was no mutation in the coding region between various haplotypes. The bar plot showed the relative expression levels of *CsaV3_1G012520* (**G**) and *CsaV3_1G013060* (**I**) genes. **H** The percentage of Hap1, Hap2, Hap3 and Hap4 genotypes in *CsaV3_1G012520* within the four ecotypes. **J** The percentage of Hap1, Hap2 and Hap3 genotypes in *CsaV3_1G013060* within the four ecotypes. The proportion was listed as follow. Significance was assessed through the two-sided Student’s *t*-test in (**C**, **D**, **G** and **E**). Statistical significance levels were denoted as *P < 0.05 and **P < 0.01. Mean values ± standard error (SE) was presented for data in G and I

Within the interval defined by locus *gLTT1.1* (Fig. 5A), fourteen orthologous genes associated with LT stress (Table S9; Fig. 5B) were identified. They encoded: (a) zinc finger proteins (Wang et al. 2019a), i.e., *CsaV3_1G0122*30, *CsaV3_1G012850*, *CsaV3_1G012940*, *CsaV3_1G013000*, *CsaV3_1G013060*, *CsaV3_1G013100*, and *CsaV3_1G013260*; (b) peroxidases (Wang et al. 2022b; Zhou et al. 2016; Song et al. 2023), i.e., *CsaV3_1G012650;* (c) a mitogen-activated protein kinase (Li et al. 2017; Yang et al. 2010), i.e., *CsaV3_1G012740*; (d) a bHLH transcription factor (An et al. 2020; Yang et al. 2023; Zhang et al. 2023; Wu et al. 2022), i.e., *CsaV3_1G012350*; (e) a NAC transcription factor (Diao et al. 2020), i.e., *CsaV3_1G012610*; and two ethylene-responsive transcription factors (Mizoi et al. 2012; Bai et al. 2021; Xu et al. 2023), i.e., *CsaV3_1G012520*; (f) GUB_WAK_bind domain-containing protein; (g) a NAC transcription factor (Tang et al. 2022), i.e., *CsaV3_1G010400*; and (h) TCP transcription factor (Tian et al. 2022), i.e., *CsaV3_1G012680*.

To determine if any of these genes may be causal for cold tolerance, the various haplotypes derived from the promoter, intron, and exons were aligned. Five genes ((*CsaV3_1G012350* (Fig. S3A), *CsaV3_1G012520* (Fig. 5C), *CsaV3_1G012850* (Fig. S3B), *CsaV3_1G013060* (Fig. 5D) and *CsaV3_1G013000* (Fig. S3C)) showed DNA polymorphisms between various haplotypes, while haplotypes of the other nine genes were identical (Figs. S3D-I; Table S10). *CsaV3_1G012350* had 21 polymorphisms in the promoter between hap1 and hap5, and another two SNPs within exons were found in *CsaV3_1G012350*. These exons contained two G-to-A/C-to-G synonymous substitutions in the first and second exon (Fig. S4A). For *CsaV3_1G012520*, 29 mutations were located in the promoter, and two SNPs within the exon would result in a synonymous mutation between hap1/4 and hap2/3 (Fig. 5E). There were 15 mutations in *CsaV3_1G012850* – two in the promoter, 11 in different introns and two in the exon between hap1/3 and hap2. Of these, two SNPs were predicted to lead to synonymous substitutions (Fig. S4B). There was one mutant site in the *CsaV3_1G013100* promoter between hap1 and hap2 (Fig. S4C; Table S10). Fourteen polymorphisms (13 in the promoter and one in the intron) of *CsaV3_1G013060* were detected between hap1 and hap3 (Fig. 5F; Table S10).

The expression level of the five candidate genes tested at *gLTT1.1*, i.e., *CsaV3_1G012350, CsaV3_1G012520, CsaV3_1G012850, CsaV3_1G013100* and *CsaV3_1G013060*, differed between the haplotypes. For *CsaV3_1G012520*, Hap2 individuals were more LT-sensitive than those with Hap1/3/4 (Fig. 5C). After 14 d, the expression of *CsaV3_1G012520* was higher in susceptible lines ‘R51’ (Hap1) and ‘CG84’ (Hap3) compared to the susceptible line ‘CG31’ carrying Hap2 (Fig. 5G). Interestingly, the tolerant Xishuangbanna type only included the tolerant Hap3 haplotype (Fig. 5H). For *CsaV3_1G013060*, Hap1 genotypes were LT-resistant line while the Hap3 were sensitive (Fig. 5D). The expression of *CsaV3_3G013060* in the LT-resistant line 'CG84' with Hap3 was significantly higher than in the susceptible lines (‘R59’ and ‘CG31’) carrying Hap1 at 21 d, 28 d, 35 d, 56 d after sowing (Fig. 5I). Coincidentally, the tolerant Xishuangbanna also only possessed the tolerant Hap3 (Fig. 5J). The other three genes (*CsaV3_1G012350*, *CsaV3_1G012850*, *CsaV3_1G013100*) had no significant differences in expression (Fig.S5A-5C). Therefore, we hypothesize that *CsaV3_1G012520* and *CsaV3_3G013060* play a role in governing LT tolerance in adult cucumber plants.

**Fig. 6:**
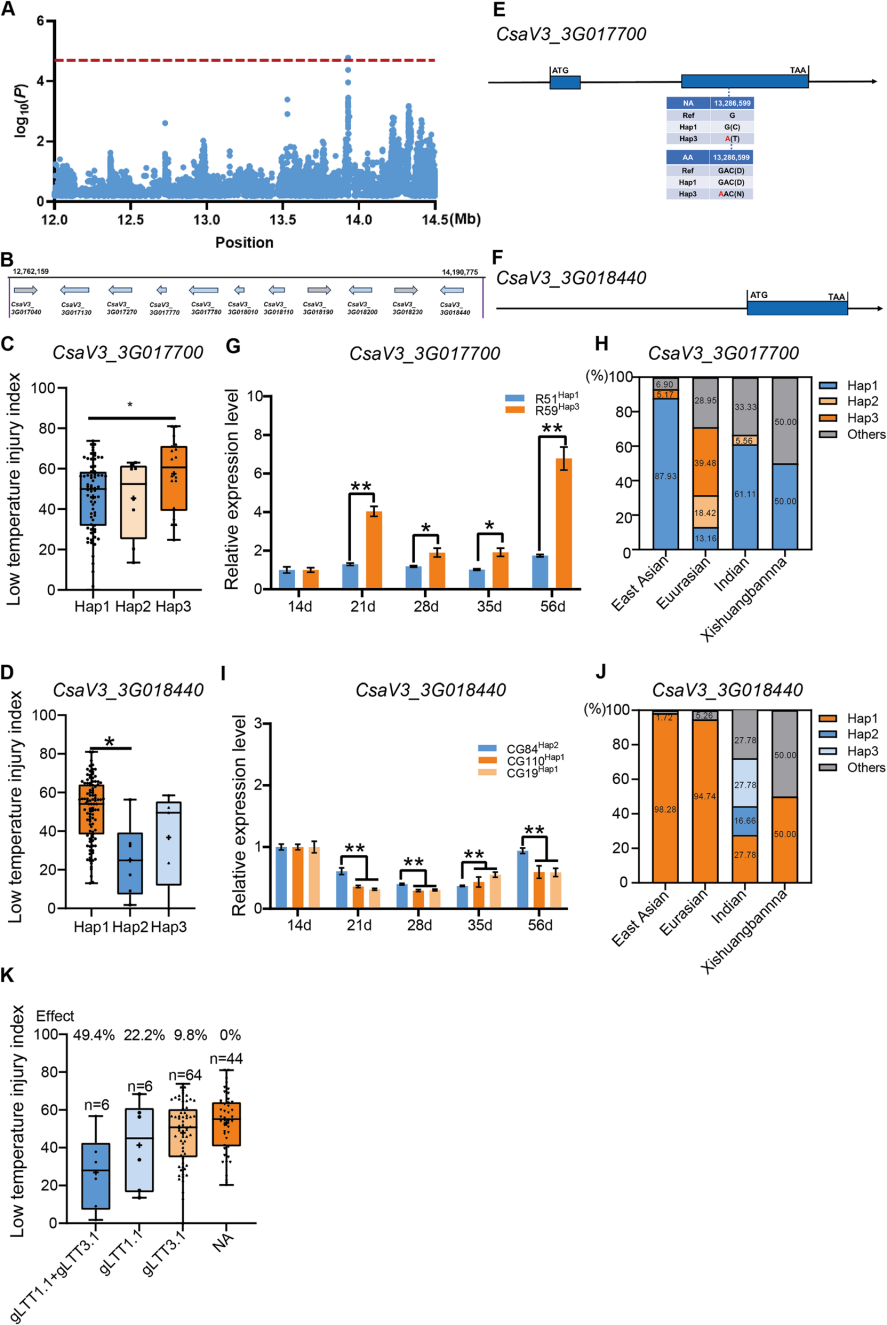
Assessment of *CsaV3_3G017700* and *CsaV3_3G08440* as candidate genes associated with LT-tolerance in adult cucumber at the *gLTT3.1* locus. **A** A Manhattan plot focused on *gLTT3.1* (top) is displayed, with the red horizontal dashed line representing the GWAS significance threshold set at -log10(*P*) = 4.5. **B** Within the *gLTT3.1* candidate region, eleven functionally annotated genes related to abiotic stress were predicted. Each gene was shown as an arrow, which also indicated gene directionality. **C-D** Box plots showing the LTII distributions of accessions carrying distinct haplotypes of the (**C**) *CsaV3_3G017700* and (**D**) *CsaV3_3G018440* genes. **E** Sequence analysis of *CsaV3_3G017700*. A base substitution (G/A) is predicted to lead to a (Asp → Asn) substitution (**F**) Sequence analysis of *CsaV3_3G018440*, did not reveal mutations in the coding region. Bar plot showed the relative expression levels of the (**G**) *CsaV3_3G01770* and (**I**) *CsaV3_3G018440* genes. The percentage of Hap1, Hap2 and Hap3 genotypes in (**H**) *CsaV3_3G017700* and (**J**) *CsaV3_3G018440* within four ecotypes. **K** A box plot showing LTII effects of low temperature-sensitive allelic combinations, representing the accessions categorized according to all of the different allelic combinations found in all the resequenced accessions. The number of accessions for each category is shown above each column, and the proportions are also listed. Significance was assessed through the two-sided Student’s *t*-test in (**C**, **D**, **G** and **I**). Statistical significance levels were denoted as *P < 0.05, **P < 0.05. Mean values ± standard error (SE) was presented for data in **G** and **I**

The candidate region associated with locus *gLTT3.1* spans from 12,762,159 bp to 14,190,775 bp (Fig. 6A). Within this region, there were 148 annotated genes, eleven of which are implicated in abiotic stress pathways (Table S9; Fig. 6B). Six genes (*CsaV3_3G017130*, *CsaV3_3G017700, CsaV3_3G017780*, *CsaV3_3G018010*, *CsaV3_3G018110, CsaV3_3G018440*) encoded zinc finger proteins, many of which have reported roles in abiotic stress (Chen et al. 2018; Liu et al. 2022b; Wang et al. 2019c). *CsaV3_3G017040* and *CsaV3_3G018200* encode a MYB transcription factor, which has a role in plant cold response (Xie et al. 2018; Zhang et al. 2020). *CsaV3_3G018190* encodes a cinnamyl alcohol dehydrogenase (Liu et al. 2014). *CsaV3_3G017270* and *CsaV3_3G018230* encode a mitogen-activated protein kinase which is involved in a cold stress pathway (Zhao et al. 2017; Zhang et al. 2017).

Eight of the eleven genes (See Fig.S3J-3P) described above consisted of a single haplotype. The remaining three genes, i.e., *CsaV3_3G017700, CsaV3_3G018010* and *CsaV3_3G018440* showed multiple haplotype (Fig. 6C,6D; Fig.S3J). *CsaV3_3G017700*, had 37 mutations located in the promoter. There was also one SNP (G/A) located in the second exon, that is predicted to lead to an Asp → Asn substitution (Fig. 6E). Examination of *CsaV3_3G018010*, identified one SNP in the promoter, and in the exon, an A-to-C SNP, which is predicted to cause a non-synonymous Ile → Leu substitution (Fig.S4D). We identified three SNPs in *CsaV3_3G018440* all localized to the promoter (Fig. 6F; Table S10).

The LTII of individuals with the *CsaV3_3G017700* Hap1 genotype were significantly lower than that of those that were Hap3 (Fig. 6C). Interestingly, *CsaV3_3G017700* expression was induced to a higher level at 21 d after cold exposure in the susceptible line ‘R59’ harboring Hap3, while conversely, its expression decreased in the resistant line ‘R51’ carrying Hap1. Interestingly, the relative expression level of *CsaV3_3G017700* in ‘R59’ was more than four times higher than that of ‘R51’ at 35- and 56 d after sowing (Fig. 6G). Meanwhile, LT-sensitive Hap3 accessions not happened in India and Xishuangbanna ecotype (Fig. 6H). A significant difference in LTII was found between Hapl and Hap2 (Fig. 6D). Transcript profile of *CsaV3_3G018440* was higher in ‘CG84’ harboring Hap2 than that of ‘CG110’ and ‘CG19’ harboring Hap1 before (14 d, 21 d, 35 d after sowing) and after (56 d after sowing) exposure to cold (Fig. 6I). The LT sensitive- East Asian and Eurasian ecotypes had a high proportion (98.28% and 94.74%) of LT-sensitive accessions harboring LT-sensitive haplotype Hap1. The expression of *CsaV3_3G018060* did not exhibit a significant difference (Fig.S5D). Therefore, we proposed that both *CsaV3_3G017700* and *CsaV3_3G018440* were putative candidates in the LT response of cucumber at the adult stage.

To further identify the relationship between *gLTT1.1* and *gLTT3.1*, the pyramiding effects of the haplotypes at the *gLTT1.1* and *gLTT3.1* loci were analyzed by comparing the LTII data among accessions carrying multiple resistant-LT allelic combinations. Interestingly, accessions harboring more LT-resistant haplotype (e.g., Hap3 of *CsaV3_1G013060* in *gLTT1.1* or Hap1 of *CsaV3_3G017700* in *gLTT3.1*) invariably showed relatively higher LT-tolerance (Fig. 6K); six accessions harboring both LT-resistant haplotypes, showed the highest LT-tolerance, with a 49.4% higher LTT based on the LTII (Fig. 6K). Above all, these results provided target alleles for breeding LT-tolerant cucumber by combining multiple resistant haplotypes.

## Discussion

Previous studies showed that LT-tolerance in cucumber seedlings is controlled by multiple genes (Dong et al. 2019) and in some cases, a single gene (Kozik and Wehner 2008). In this study, 120 cucumber accessions and a RIL population of 140 adult individuals, derived from ‘CsIVF0106’ (LT-sensitive) and ‘CsIVF0168’ (LT-tolerant) were scored using our LTII. The LTII was continuously distributed, which indicated that the LT-tolerance we assessed in the cucumber adult plants in this study, was controlled by multiple genes. This is the first study of the inheritance pattern of LT-tolerance performed at cucumber adult stage.

Cucumber research has primarily concentrated on seedlings rather than mature plants, resulting in few identified QTLs or genes associated with LT-tolerance. Our study aimed to pinpoint candidate genes responsible for LT-tolerance in adult cucumber plants. This study was the first report for candidate genes underlying LT-tolerance in cucumber adult plants, combining GWAS and QTL analysis. We used a panel of 120 cucumber accessions to perform a GWAS analysis for LT-tolerance. Two loci: one on Chr.1 (*gLTT1.1*) and another on Chr.3 (*gLTT3.1*) were identified. Meanwhile, using a RIL population and 156 markers, we mapped two robust loci—*qLTT1.2* and *qLTT3.1* associated with LT-response. Interestingly, *qLTT1.2* and *gLTT1.1*, and *qLTT3.1* and *gLTT3.1* were co-located. Candidate genes were identified by examining haplotype polymorphisms among stress-related genes at these QTLs, and then, by determining if these genes showed a differential response to LT stress in the sensitive and tolerant haplotypes. In this way, we successfully narrowed the genes at *gLTT1.1* and *gLTT3.1*, respectively*,* to two candidates. Collectively, these results suggested that the two loci were reliable and stable, and importantly, that these genes may functionally regulate LT response in adult cucumber plant.

**Fig. 7:**
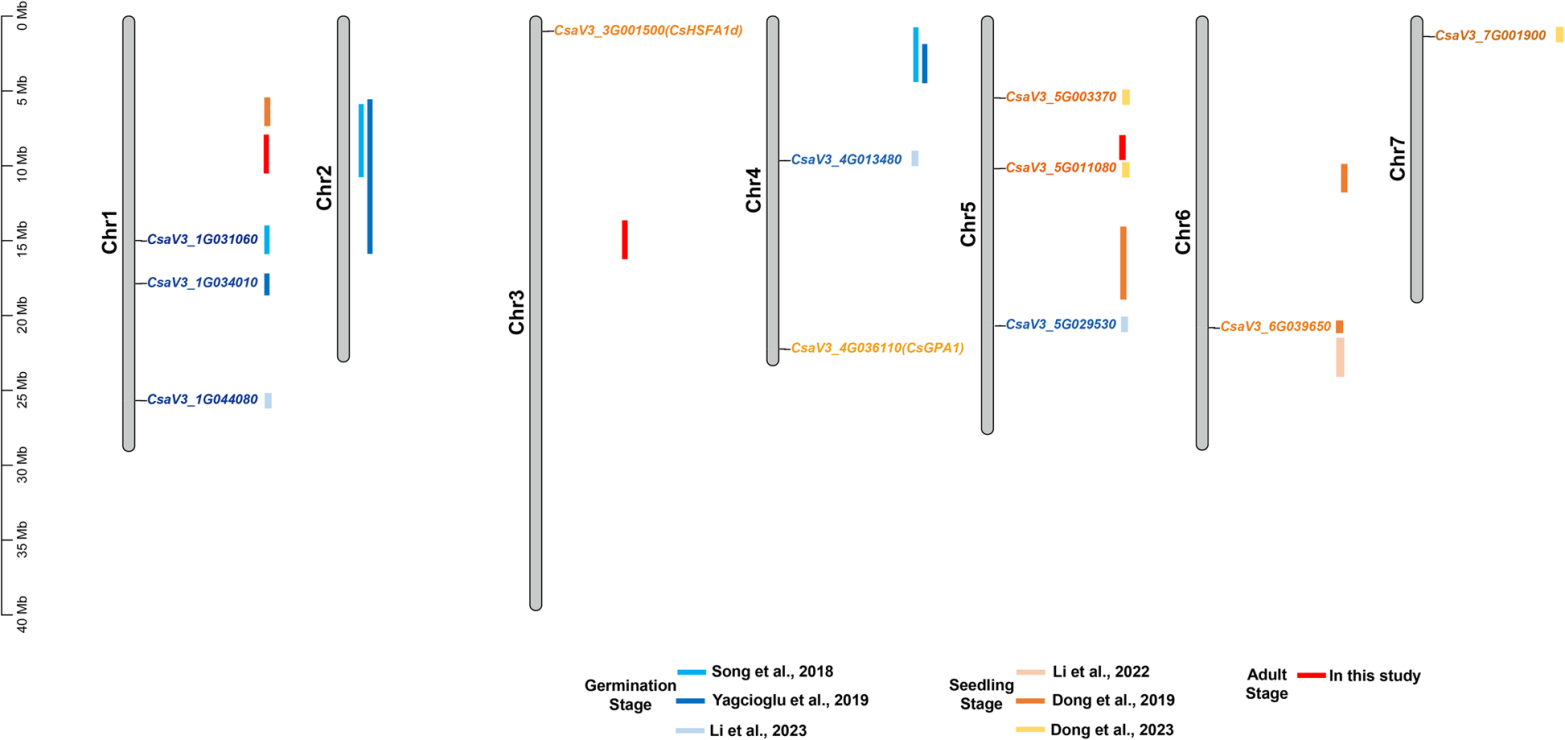
Locations of QTLs for cucumber LT-tolerance reported in this study and previous studies

Many QTLs and genes for LT-tolerance in cucumber during germination or the seedling stage have been discovered by traditional mapping or by GWAS (Fig. 7). By using different evaluation indices for LT stress, i.e., relative germination-rate, -energy, -index and -radical length, three loci on Chrs.1, 2 and 4 were mapped by QTL analysis, and three loci on Chrs.1, 4 and 5 were detected by GWAS (Yagcioglu et al. 2019; Song et al. 2018; Li et al. 2022b, 2023b). Meanwhile, Li et al. (2022a) detected one locus for chilling stress on Chr.6 and Dong et al. (2019) found three loci on Chrs.1 and 6 by long-term LT stress. Dong et al. (2019) mapped the minor locus *qLTT1.1* to a 1.02-Mb region on Chr.1 by markers SSR02810 and SSR16811, stretching from 5,870,967 bp to 6,891,308 bp. In this study, the *gLTT1.1* locus in adult cucumber plant was mapped to Chr.1 region from 6,460,155 bp to 8,611,538 bp, which overlapped with the region mapped by Dong et al. (2019). The QTL on Chr.3 we identified was novel, as it had not been previously described. Interestingly, we found that the East Asian accessions were LT-sensitive at the adult stage (Fig. 4C), while the opposite result was observed by Wang et al (2019a, b, c) where the East Asian accession were LT-tolerant at the seedling stage. Therefore, LT-tolerance in cucumber during germination, and at the seedling and adult stages may be govern by distinct genetic mechanisms.

The mechanisms involved in plant response to LTs are intricate; they include signal perception, transduction and regulation, across the cytomembrane, cytoplasm and cell nucleus, respectively (Ding et al. 2019; Kidokoro et al. 2022). The regulation of LT response involves multiple factors, e.g., bHLH (Xu et al. 2014; Kidokoro et al. 2022; Zhang et al. 2023), NAC (Zhuo et al. 2018; Diao et al. 2020), MYB (Xie et al. 2018; Jiang et al. 2022), and ERF (Li et al. 2023a; Wang et al. 2021; Mizoi et al. 2012). Moreover, other factors are also important for plant LT-response including phytohormones like ethylene (Huang et al. 2023); kinases (Zhao et al. 2017; Li et al. 2017); reactive oxygen species (ROS) (Wang et al. 2023a; Mittler et al. 2022), and an E3 ubiquitin protein ligase (Wang et al. 2022a). Twenty-five genes potentially related to LT-tolerance were identified within the delimited GWAS and QTL regions, and four were identified as potential causal genes. These candidates include, *CsaV3_1G012520*, an ethylene-responsive transcription factor and *CsaV3_1G013060*, a RING/U-box superfamily protein within the *gLTT1.2* locus (Fig. 5), and *CsaV3_3G017700* and *CsaV3_3G017040* which are both RING-type E3 ubiquitin ligase within *qLTT3.1* (Fig. 6). These genes either have haplotype differences or vary in expression variations when compared between resistant- and sensitive- accessions of various haplotypes.

*CsaV3_1G012520* encodes an ethylene-responsive transcription factor (ERF), which has known roles in plant LT response in a variety of species. Orthologues of ERF positively regulates cold stress including *MdERF1B* and *MdERF2* in apple (*Malus domestica*) (Wang et al. 2021), *PtrERF9* in *Poncirus trifoliata* (L.) Raf. u (Zhang et al. 2022), *ERF.D4* in tomato (Klay et al. 2018), and *VaERF057* in grapevine (Sun et al. 2016). The other candidate gene at *gLTT1.2* was *CsaV3_1G013060*, a RING/U-box superfamily protein, which in *Arabidopsis* encodes a zinc finger RING family protein. There have been no published data on this specific gene isoform in *Arabidopsis* and its precise function in responding to abiotic stress is yet to be explored.

Candidate gene *CsaV3_3G017700*, a RING-type E3 ubiquitin ligase, is homologous to a protein with a RING/U-box and TRAF-like domain. Candidate gene *CsaV3_3G018440*, also a RING-type E3 ubiquitin ligase, is homologous to plant U-box 26 protein. These two RING ubiquitin transferases are implicated in the cold stress response of plants. Homologues of *CsaV3_3G018440*, namely the E3 ubiquitin ligase proteins OsPUB2 and OsPUB3, function as positive regulators in rice cold stress mechanisms (Byun et al. 2017). Another E3-ubiquitin ligase *HOS1* (Osmotically Responsive Gene 1) interacts with the INDUCER OF CBF EXPRESSION (ICE1) protein to manage cold stress in rice and *Arabidopsis* (Lourenço et al. 2013; Dong et al. 2006; Lee et al. 2001). Finally, *OsSRFP1*,encoding an E3 ubiquitin ligase, acts to suppress cold stress response in rice via transcriptional and post-translational activity (Fang et al. 2015).

To summarize, by combining GWAS and QTL mapping, we discovered overlapping loci related to LT-tolerance in cucumber at the adult stage. Further, we hypothesize that four genes: *CsaV3_1G0125210*, *CsaV3_1G013060*, *CsaV3_1G017700*, and *CsaV3_3G018440* are candidates for determining the LT response in adult cucumber plants. This assessment is based on differences in their relative expression levels among accessions with contrasting haplotypes. Nevertheless, the functional role of these genes in LT tolerance remains to be elucidated through experiments involving overexpression or suppression in cucumber via gene transformation techniques. Overall, our study will serve as a valuable resource for understanding the mechanisms underlying LT tolerance and for breeding cucumber varieties with enhanced cold tolerance.

## Materials and methods

### Plant materials

The 120 cucumber accessions utilized in the GWAS analysis (Table S3) were derived from various geographical origins. For the RIL population, two inbred lines, ‘CsIVF0106’ and ‘CsIVF0168’, were used as the parentals to develop the F_1_ population. ‘CsIVF0106’, is a northern European greenhouse cucumber-type, is tolerant to LT stress, while ‘CsIVF0168’ is a northern Chinese fresh-market type with LT sensitivity (Fig. S2A). The F_2_ were generated by self-pollinating the F_1_, and 140 RIL individuals were selected using the single seed descent method.

### Phenotypic data collection

The 120 accessions, RILs parental lines (‘CsIVF0106’, ‘CsIVF0168’), F_1_ individuals, and the RIL population were evaluated two rounds of evaluation in the plastic greenhouse at Shouguang, Shandong, China (36^◦^88′N,118^◦^73′E) in winter 2021 (2021_W), 2022 (2022_W) and 2023 (2023_W), respectively. Plants encountered a naturally occurring LT stress as emerging leaves were yellowed and dried at 86, 96, 102 days after sowing in 2021_W, 2022_W and 2023_W, respectively (Table S1). Detailed real-time temperature logging was done and the data was listed in Table S1 and Fig. S1. Each test set contained three replicates following a randomized block design, with each replicate comprising five individual plants.

To evaluate symptom severity, a LTII was used. The grading of LT injury was determined by assessing the ratio of yellowing to dryness of the fourth to sixth functional leaves of each plant from top to bottom (Fig. 1A): Grade 0: leaves were dark-green; no visible symptoms; Grade 1: leaves were green; slight withering at the leaf’s edge; Grade 3: leaves were light-green; withered area was < 50%; Grade 5: leaves were yellow, the withered area was 50–75%. Grade 7: leaves were dark-yellow; the withered area was > 75%; Grade 9: All leaves were withered, and some plants died. The calculation of LTII involved the formula: LTII = (0 × S0 + 1 × S1 + 3 × S3 + 5 × S5 + 7 × S7 + 9 × S9)/(N × 9), where 'S' represents the number of plants in each grade, and 'N' represents the total number of plants assessed. The LTII of each line was the average of the LTII in the number of biological replicates in each experiment.

### GWAS analysis

The cucumber accessions were re-sequenced (Qi et al. 2013; Liu et al. 2022a). Association analysis was conducted using a linear mixed model implemented in the program factored spectrally transformed linear mixed model (FaST-LMM), incorporating an estimated relatedness matrix as a covariate (Haas et al. 2003). The GWAS analysis was conducted, and a genome-wide *p*-value was obtained. The threshold for genome wide significance was -log_10_(*P*) value of 4.5. SNP (single nucleotide polymorphism) genotyping, assessment of population structure, and determination of relative kinship for mapping were identified (Qi et al. 2013). Manhattan plots were generated using the ‘qqman’ package with in the R environment, following the method described by Turned (2014).

### QTL mapping

A genetic map with 156 markers was developed using a RIL population (Shi et al. 2018). The phenotyping of the cucumber for LT-tolerance was assessed in winter of 2021 and 2022, (described as 2021_W and 2022_W respectively) (Table S11), and QTL analysis of the data was achieved using software QTL IciMapping 4.1 (CAAS, Beijing, China (Meng et al. 2015)). A linkage map was developed, and the genome wide logarithm of the odds (LOD) for QTLs were identified as default in QTL IciMapping 4.1. QTLs were designated following a specific naming convention, which consists of an abbreviation representing the trait (e.g., LT-tolerance—LTT), the chromosome (Chr.) number and the locus number (Wan et al. 2010). For instance, *qLTT1.2* indicates the second QTL on cucumber Chr.1, *qLTT3.1* signifies the first QTL on cucumber Chr.3 and so on.

To further narrow the QTL region, Insertion and Deletion (InDel) markers were generated. The Chinese Long cucumber ‘9930’ reference genome was utilized along with resequencing data from 'CsIVF0106' and 'CsIVF0168'. Primers were designed using DNAMAN 7 software based on the 5 ~ 10 bp differences observed between the parental lines (Woffelman 2004). All polymorphic markers (Table S12) were selected and used to construct linkage genetic map.

### Functional prediction and haplotype analysis of candidate gene

The chromosomal positions and annotation of the candidate genes were obtained based on the ‘Cucumber genome V3’. The candidate genes in the regions from both the GWAS and the QTL mapping were identified as follow: first, genes involved in abiotic stresses were obtained using the TAIR and (GO) databases. Secondly, based on the cucumber resequencing data of 120 accessions, the haplotypes in the promoter or exons of various genes, that could be differentiated among the various accessions studied were identified. Thirdly, the transcript profiles of these candidate genes were compared at various time points between sensitive- and tolerant-accessions harboring the corresponding haplotypes. Ultimately, the identification of candidate genes was refined through an analysis of haplotype distribution among ecotypes.

### Extraction of RNA and qrt-PCR

Three tolerant lines (‘R51’, ‘CG84’, ‘R77’) and four sensitive lines (‘CG110’, ‘CG31’, ‘CG19’, ‘R59’) were sown on 15th August in 2022_W. The young leaves were collected at different days after sowing, i.e., 14 d, 21 d, 28 d, 35 d and 56 d. Plants at 56 d were exposed to LT stress, all others were not. Each experiment was conducted with three biological replicates. Total RNA extraction was performed using the RNeasy Plant Mini Kit (TaKaRa, Kyoto, Japan), and first-strand cDNA synthesis was carried out using the PrimeScriptTM Reagent Kit with gDNA Eraser (TaKaRa, Kyoto, Japan). Quantitative real-time PCR (qRT-PCR) was performed using SYBR Premix ExTaqTM II (TaKaRa, Kyoto, Japan) following the manufacturer's instructions. PCR amplification conditions were set according to the manufacturer's protocol. The reference gene *Actin1* (*Csa3G806800*) was used for normalizing gene expression values (Wan et al. 2010). Relative expression levels of the candidate genes were determined using the 2^−ΔΔCt^ method (Livak and Schmittgen 2001). Gene specific primers were designed using DNAMAN (Woffelman 2004), and all primers used are provided in Table S12.

### Statistical analysis

Statistical analysis was conducted to identify significant differences among different accessions using a two-tailed, two-sample Student’s t-test, with significance levels set at *p* < 0.05 or *p* < 0.01, performed using Excel 2021. Charts were drawn using GraphPad Prism 9.0 (GraphPad, San Diego, CA, USA). Cluster analysis was conducted in R software, utilizing both the sum of squares of dispersion (Ward) method and the Euclidean distance method (Turner 2014).

## Conclusion

The LT-tolerance of 120 cucumber accessions and 140 recombinant inbred lines (RILs) derived from a biparental cross were evaluated in naturally occurring LT environments at adult stage over two years. A total of 18 accessions were evaluated as highly LT tolerant, and two loci (*gLTT1.1* and *gLTT3.1*) exhibited strong signals in both environments were identified via GWAS. Genetic analysis using the RIL population revealed that the LT-tolerance in the adult cucumber plants was a multigenic quantitative trait. In addition, two QTLs—*qLTT1.2* on Chr.1, and *qLTT3.1* on Chr. 3, were discovered in all tests, which were co-localized with the loci (*gLTT1.1* and *gLTT3.1*) detected via GWAS. Then *qLTT1.2* was delimited to a 1.24-Mb region and *qLTT3.1* was narrowed to a 1.43-Mb region, and four candidates: *CsaV3_1G012520* (an ethylene-responsive transcription factor) and *CsaV3_1G013060* (a RING/U-box superfamily protein) in *gLTT1.1*, and two RING-type E3 ubiquitin transferases at *CsaV3_3G018440* and *CsaV3_3G017700* in *gLTT3.1* that may regulate LT-tolerance in adult cucumber were identified. These findings therefore provide a solid foundation for the identification of LT-tolerant genes and the molecular breeding of cucumber with LT-tolerance.

## Supplementary information

Supplementary Material 1.

## Data Availability

The datasets mentioned in this study are available in a published article and various online repositories. Details regarding the repository/repositories and corresponding accession number(s) can be found in the Supplementary Material section.

## Abbreviations

GWAS: Genome-wide association studies
RILs: Recombinant inbred lines
QTL: Quantitative trait locus
LT: Low temperature
LTII: Low temperature injury index
SNP: Single nucleotide polymorphism
CII: Cold injury index
JAZ: Jasmonic acid
LTT: Low temperature tolerance
LOD: Logarithm of odds
