# Supplementary material for: Candidate genes associated with low temperature tolerance in cucumber adult plants identified by combining GWAS & QTL mapping
Source: Stress Biol. 2024 Dec 11;4(1):53. doi: 10.1007/s44154-024-00191-9 (PMC11631831; doi:10.1007/s44154-024-00191-9)
Supplement: Supplementary file 1 — Supplementary Material 1. [file 44154_2024_191_MOESM1_ESM.docx]

**Candidate genes associated with low temperature tolerance in cucumber adult plants identified by combining GWAS & QTL mapping**

Caixia Li^a,#^, Shaoyun Dong^a,#^, Diane M Beckles^b^, Xiaoping Liu^a^, Jiantao Guan^a^, Zaizhan Wang^a^, Xingfang Gu^a^, Han Miao^a,*^, Shengping Zhang^a,*^

^a^ *State Key Laboratory of Vegetable Biobreeding*, *Institute of Vegetables and Flowers, Chinese Academy of Agricultural Sciences, Beijing, 100081, China*

*^b^* *Department of Plant Sciences, University of California, Davis, One Shield Avenue, Davis, CA 95616, USA;*

^#^ These authors contributed equally to this work.

* Correspondence authors: zhangshengping@caas.cn; miaohan@caas.cn

**
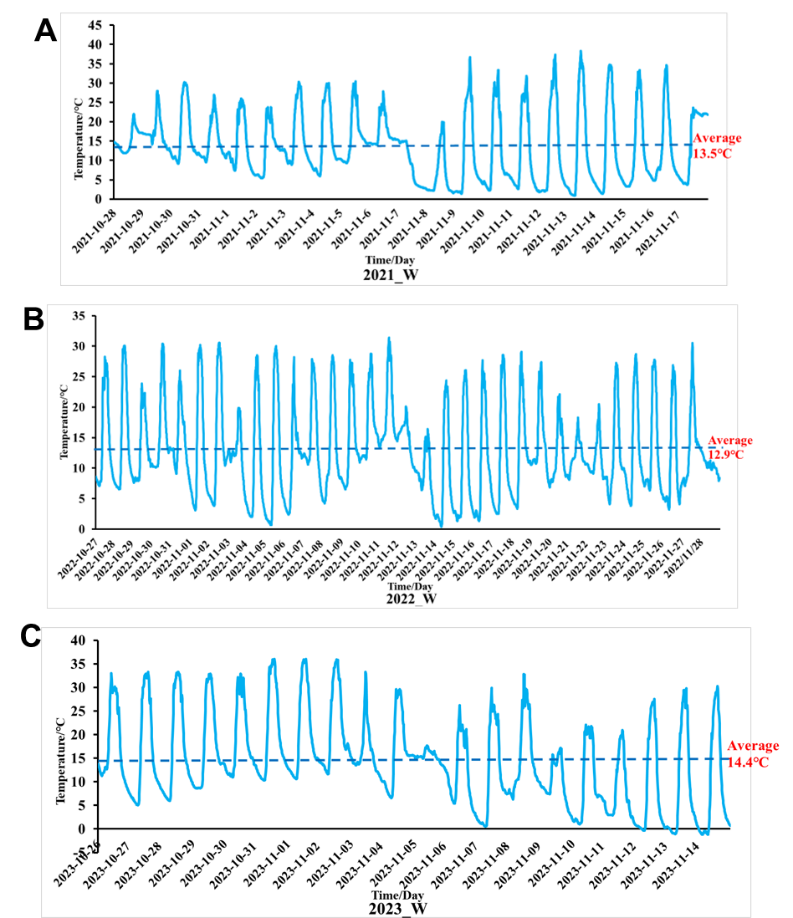
**

**Fig.S1: The temperature condition of the RILs population under natural low temperature.** The temperature in 2021_W (A), 2022_W (B) and 2023_W (C).

**
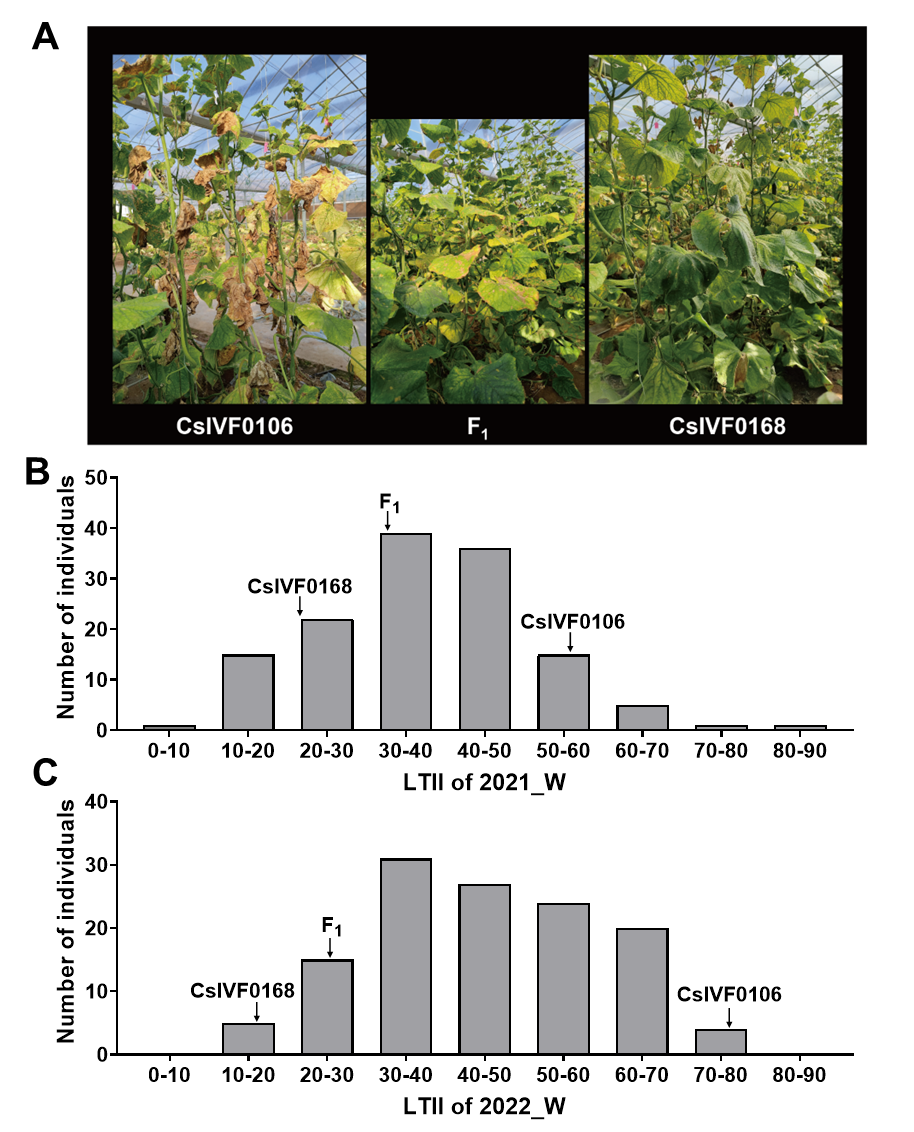
**

**Fig.S2: Phenotypic characterization of LT tolerance at cucumber adult stage.** (A) Performance of the sensitive parental ‘CsIVF0106’, the resistant line ‘CsIVF0168’, and their F_1_ hybrid progeny under LT stress. Frequency distribution of LT injury index (LTII) in RILs population in 2021_W (B) and 2022_W (C). X-axis represents the LTII; Y-axis represents the number of individuals in each LTII grades.


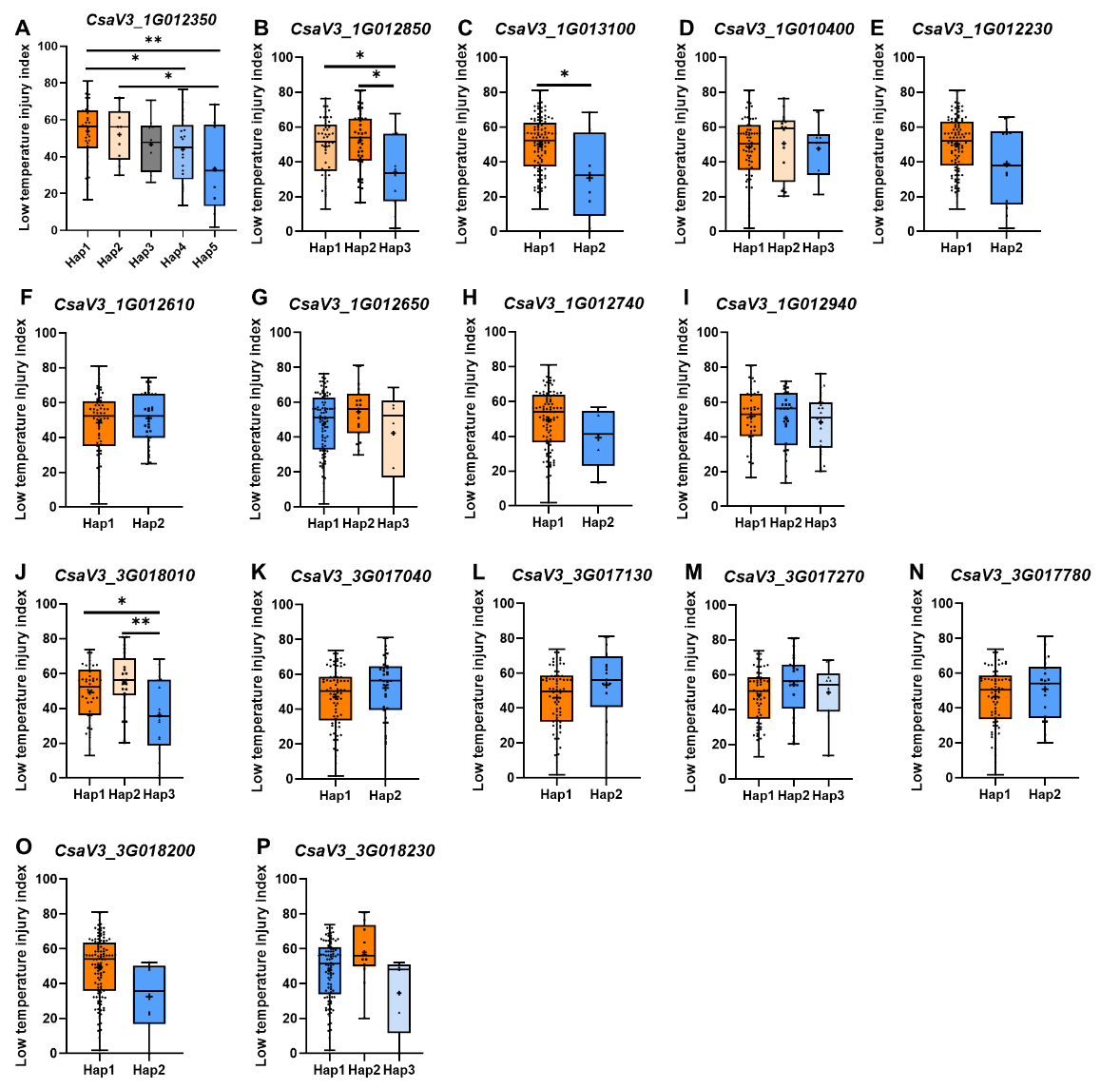


**Fig.S3: The sequence analysis of *gLTT1.2* and *gLTT3.1* candidate genes related with low temperature stress between various haplotypes.** The box plot showing the LTII distributions of accessions carrying distinct haplotypes of *CsaV3_1G012350* (A), *CsaV3_1G012850* (B), *CsaV3_1G013100* (C), *CsaV3_1G010400* (D), *CsaV3_1G012230* (E), *CsaV3_1G012610* (F), *CsaV3_1G012650* (G) , *CsaV3_1G012740* (H), *CsaV3_1G012940* (I) and *CsaV3_3G018010* (J), *CsaV3_3G017040* (K), *CsaV3_3G017130* (L), *CsaV3_3G017270* (M), *CsaV3_3G017780* (N), *CsaV3_3G018200* (O), *CsaV3_3G018230* (P).


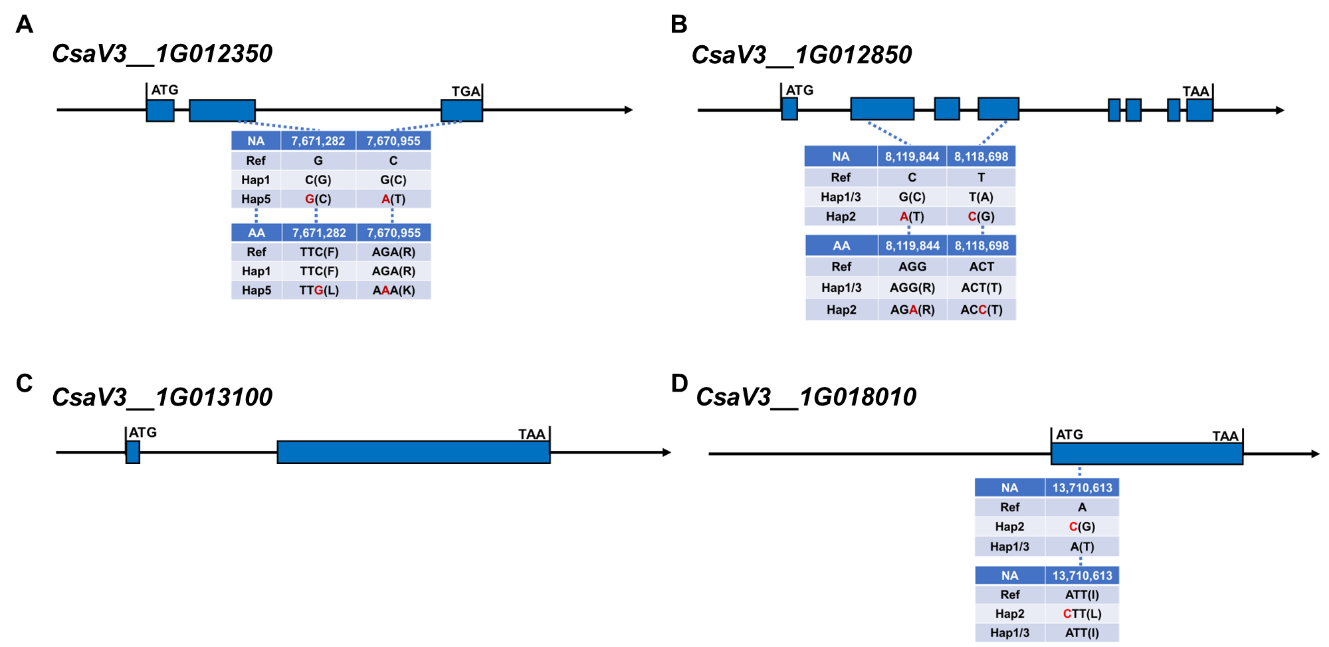


**Fig.S4: The nucleotides and amino acids sequence of *gLTT1.2* and *gLTT3.1* candidate genes between various haplotypes.** (A) Sequence analysis of *CsaV3_1G012350*. G/A, C/G polymorphism caused two Phe→Leu and Arg→Lys amino substitution. (B) Sequence analysis of *CsaV3_1G012850*. Two bases substitution (T/C, G/A) caused two nonsense mutations. (C) Sequence analysis of *CsaV3_1G013000*. There is no mutation in the coding region. (D) Sequence analysis of *CsaV3_1G018010*. The one bases substitution (A/C) caused one amino acid substitutions (Ile→Leu).


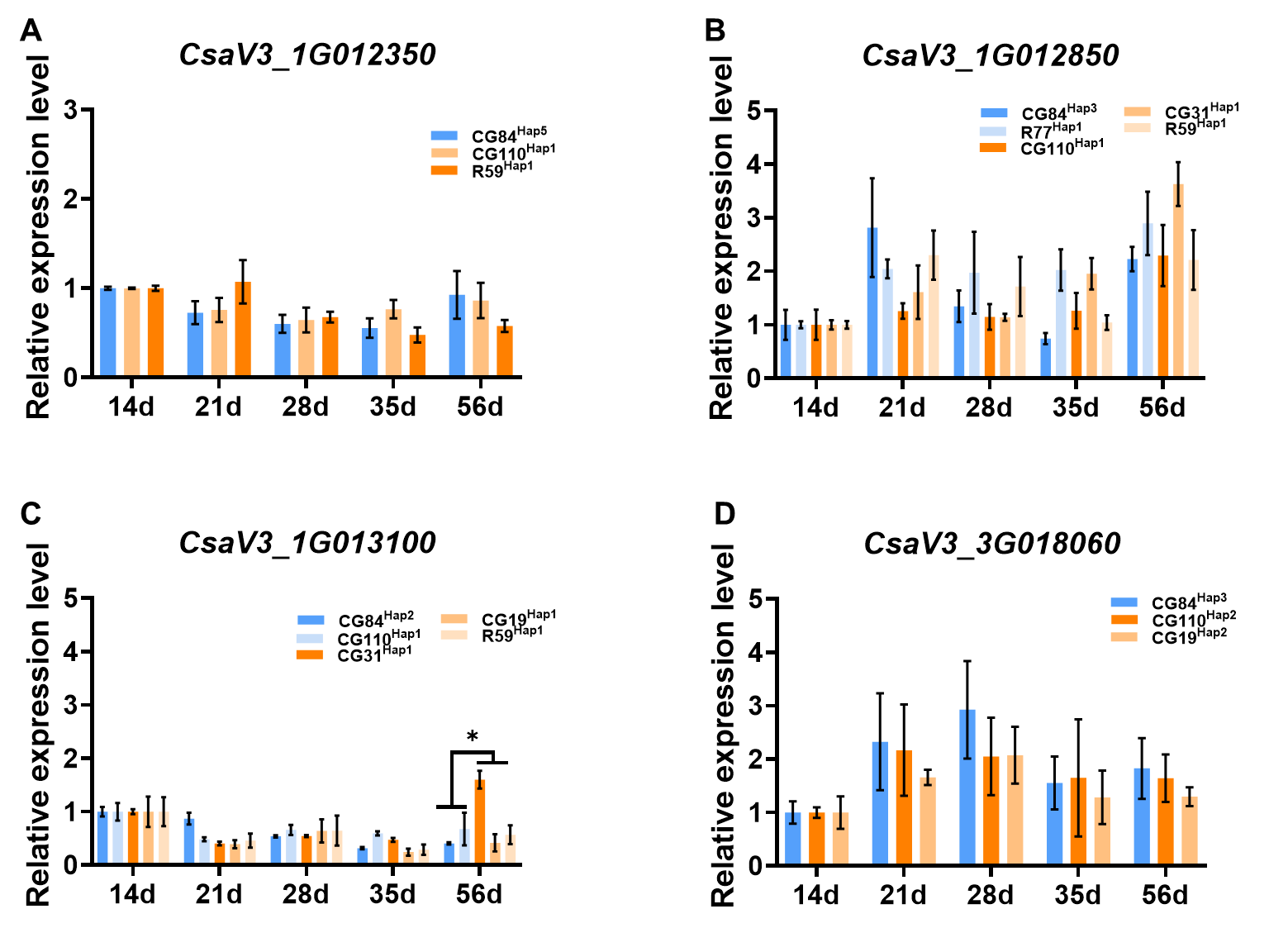


**Fig.S5: Relative expression analysis of *gLTT1.2* and *gLTT3.1* candidate genes.** Relative quantitative expression analysis of *CsaV3_1G012350* (A), *CsaV3_1G012850* (B), *CsaV3_1G013100* (C) in *gLTT1.2* and *CsaV3_3G018060* (D) in *gLTT3.1*.
